# The genome sequence of the Feathered Bright, *Incurvaria masculella* (Denis & Schiffermüller, 1775)

**DOI:** 10.12688/wellcomeopenres.19205.1

**Published:** 2023-03-30

**Authors:** William B.V. Langdon, Peter W.H. Holland, University of Oxford and Wytham Woods Genome Acquisition Lab, Darwin Tree of Life Barcoding collective, Wellcome Sanger Institute Tree of Life programme, Wellcome Sanger Institute Scientific Operations: DNA Pipelines collective, Tree of Life Core Informatics collective, Darwin Tree of Life Consortium

**Affiliations:** 1 University of Oxford, Oxford, England, UK

**Keywords:** Incurvaria masculella, Feathered Bright, genome sequence, chromosomal, Lepidoptera

## Abstract

We present a genome assembly from an individual male *Incurvaria masculella* (the Feathered Bright; Arthropoda; Insecta; Lepidoptera; Incurvariidae). The genome sequence is 552 megabases in span. Most of the assembly is scaffolded into 26 chromosomal pseudomolecules, including the assembled Z sex chromosome. The mitochondrial genome has also been assembled and is 15.3 kilobases in length.

## Species taxonomy

Eukaryota; Metazoa; Ecdysozoa; Arthropoda; Hexapoda; Insecta; Pterygota; Neoptera; Endopterygota; Lepidoptera; Glossata; Incurvarioidea; Incurvariidae; *Incurvaria*; *Incurvaria masculella* (Denis & Schiffermüller, 1775) (NCBI:txid101737).

## Background

The Incurvariidae is a small taxonomic family of moths containing only 50 to 100 species worldwide and five species in the UK. Phylogenetically, Incurvariidae lie outside the Ditrysia within the paraphyletic ‘Monotrysia’ ( Dugdale, 1974; Regier *et al.*, 2013); they therefore occupy an important evolutionary position for studies investigating the evolution of lepidopteran genetic and phenotypic characters. *Incurvaria masculella* is one of the more frequently encountered UK species of Incurvariidae, commonest in central and southern counties of England, and it is also found across northern Europe and coastal regions of Scandinavia ( GBIF Secretariat, 2022). Despite having a wingspan of just 12–16 mm, *I. masculella* is a striking moth with a yellow head, glossy purplish wings held tent-like over the body, and two sharply defined lemon-yellow diamond-shaped patches along the dorsal midline where the forewings meet. The antennae of the male are particularly notable, being large and deeply pectinate. In contrast, the antennae of the female are simple and thread-like. This sex-specific difference suggests that the unusual comb-like shape of the male antenna is likely an adaptation for increased olfactory reception in mate-finding.

The larvae of *I. masculella* are initially leaf-miners, forming circular blotch-shaped mines on leaves of the food plant, typically hawthorn *Crataegus monogyna*. The larvae then cut around the blotch and, sandwiched by leaf tissue, they drop to the ground to feed on dead leaves ( Heath & Pelham-Clinton, 1983). The moth has one generation per year in the UK with the day-flying adult on the wing in May ( NBN Gateway, 2022; Sterling & Parsons, 2018).

The complete genome sequence of *I. masculella* was sequenced as part of the Darwin Tree of Life Project based on long-read sequence data from the ilIncMasc1 male specimen from Wytham Woods, UK, scaffolded using Hi-C data from the ilIncMasc2 male specimen from Wallingford, UK. Due to the phylogenetic position of Incurvariidae, the genome sequence will be particularly useful in comparative studies investigating the evolution of lepidopteran genetic and phenotypic characters.

### Genome sequence report

**Figure 1.**
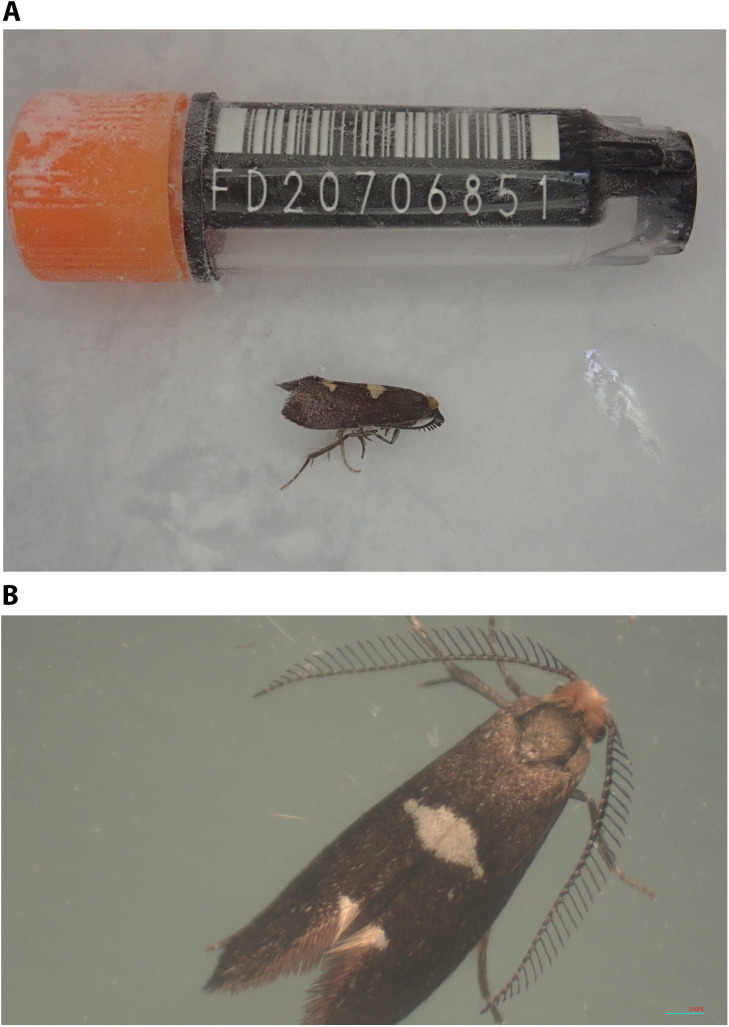
*Incurvaria* masculella. **a**) The specimen used for genome sequencing (ilIncMasc1), **b**) The specimen used for Hi-C sequencing (ilIncMasc2)

The genome was sequenced from one male *Incurvaria masculella* specimen ( Figure 1) collected from Wytham Woods, Oxfordshire, UK (latitude 51.772, longitude –1.338). A total of 32-fold coverage in Pacific Biosciences single-molecule HiFi long reads was generated. Primary assembly contigs were scaffolded with chromosome conformation Hi-C data. Manual assembly curation corrected 16 missing joins or mis-joins and removed five haplotypic duplications], reducing the assembly length by 0.77%, and decreasing the scaffold N50 by 0.75%.

**Figure 2.**
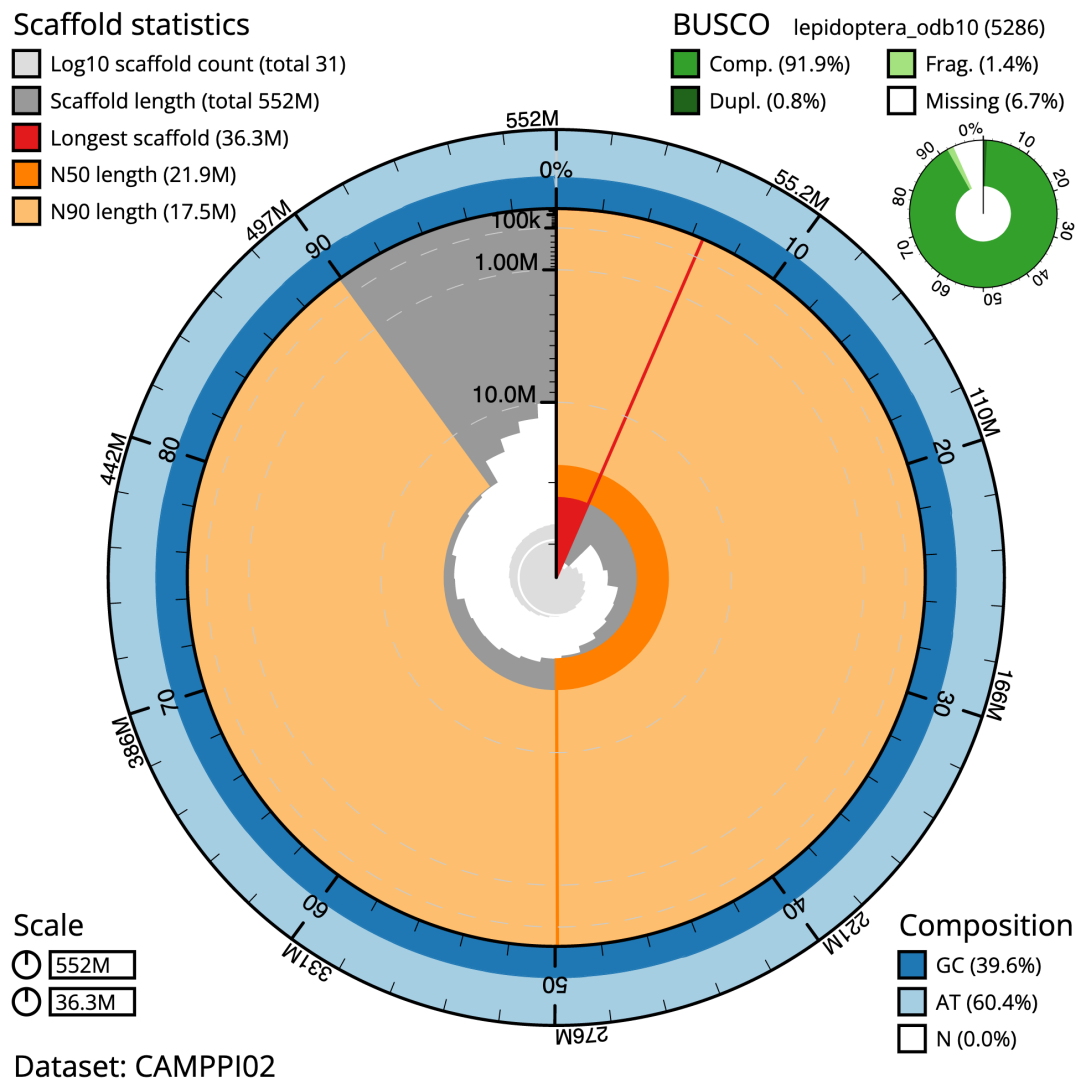
Genome assembly of *Incurvaria masculella*, ilIncMasc1.2: metrics. The BlobToolKit Snailplot shows N50 metrics and BUSCO gene completeness. The main plot is divided into 1,000 size-ordered bins around the circumference with each bin representing 0.1% of the 552,037,208 bp assembly. The distribution of scaffold lengths is shown in dark grey with the plot radius scaled to the longest scaffold present in the assembly (36,252,389 bp, shown in red). Orange and pale-orange arcs show the N50 and N90 scaffold lengths (21,889,303 and 17,498,119 bp), respectively. The pale grey spiral shows the cumulative scaffold count on a log scale with white scale lines showing successive orders of magnitude. The blue and pale-blue area around the outside of the plot shows the distribution of GC, AT and N percentages in the same bins as the inner plot. A summary of complete, fragmented, duplicated and missing BUSCO genes in the lepidoptera_odb10 set is shown in the top right. An interactive version of this figure is available at https://blobtoolkit.genomehubs.org/view/ilIncMasc1.2/dataset/CAMPPI02/snail.

The final assembly has a total length of 552.0 Mb in 31 sequence scaffolds with a scaffold N50 of 21.9 Mb ( Table 1). Most (99.97%) of the assembly sequence was assigned to 26 chromosomal-level scaffolds, representing 25 autosomes, and the Z sex chromosome. The Z chromosome was identified by similarity. Chromosome-scale scaffolds confirmed by the Hi-C data are named in order of size ( Figure 2– Figure 5; Table 2). The assembly has a BUSCO v5.3.2 ( Manni *et al.*, 2021) completeness of 91.9% (single 91.1%, duplicated 0.8%) using the lepidoptera_odb10 reference set. While not fully phased, the assembly deposited is of one haplotype. Contigs corresponding to the second haplotype have also been deposited.

**Table 1.** Genome data for *Incurvaria masculella*, ilIncMasc1.2.

Project accession data		
Assembly identifier	ilIncMasc1.2	
Species	*Incurvaria masculella*	
Specimen	ilIncMasc1	
NCBI taxonomy ID	101737	
BioProject	PRJEB55135	
BioSample ID	SAMEA10166808	
Isolate information	male; ilIncMasc1 (PacBio Hi-Fi sequencing) male; ilIncMasc2 (Hi-C)	
Assembly metrics *		*Benchmark*
Consensus quality (QV)	66.4	*≥ 50*
*k*-mer completeness	100%	*≥ 95%*
BUSCO **	C:91.9%[S:91.1%,D:0.8%], F:1.4%,M:6.7%,n:5,286	*C ≥ 95%*
Percentage of assembly mapped to chromosomes	99.97%	*≥ 95%*
Sex chromosomes	Z chromosome	*localised homologous pairs*
Organelles	Mitochondrial genome assembled.	*complete single alleles*
Raw data accessions		
PacificBiosciences SEQUEL II	ERR10033483	
Hi-C Illumina	ERR10038431	
Genome assembly		
Assembly accession	GCA_946894095.1	
*Accession of alternate haplotype*	GCA_946894085.1	
Span (Mb)	552.0	
Number of contigs	42	
Contig N50 length (Mb)	19.9	
Number of scaffolds	31	
Scaffold N50 length (Mb)	21.9	
Longest scaffold (Mb)	36.3	

* Assembly metric benchmarks are adapted from column VGP-2020 of “Table 1: Proposed standards and metrics for defining genome assembly quality” from ( Rhie *et al.*, 2021). ** BUSCO scores based on the lepidoptera_odb10 BUSCO set using v5.3.2. C = complete [S = single copy, D = duplicated], F = fragmented, M = missing, n = number of orthologues in comparison. A full set of BUSCO scores is available at https://blobtoolkit.genomehubs.org/view/ilIncMasc1.2/dataset/CAMPPI02/busco.

**Figure 3.**
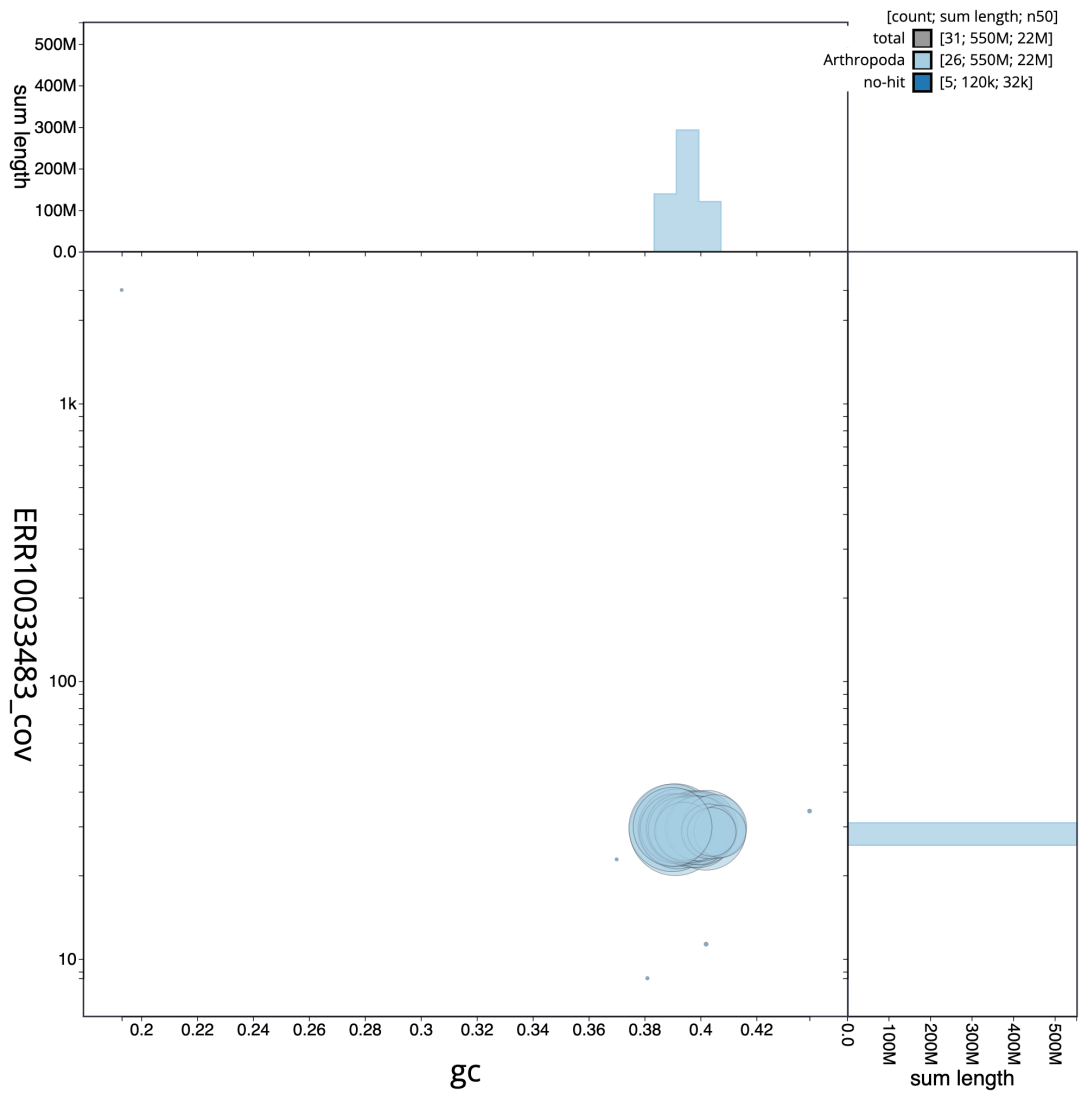
Genome assembly of *Incurvaria masculella*, ilIncMasc1.2: GC coverage. BlobToolKit GC-coverage plot. Scaffolds are coloured by phylum. Circles are sized in proportion to scaffold length. Histograms show the distribution of scaffold length sum along each axis. An interactive version of this figure is available at https://blobtoolkit.genomehubs.org/view/ilIncMasc1.2/dataset/CAMPPI02/blob.

**Figure 4.**
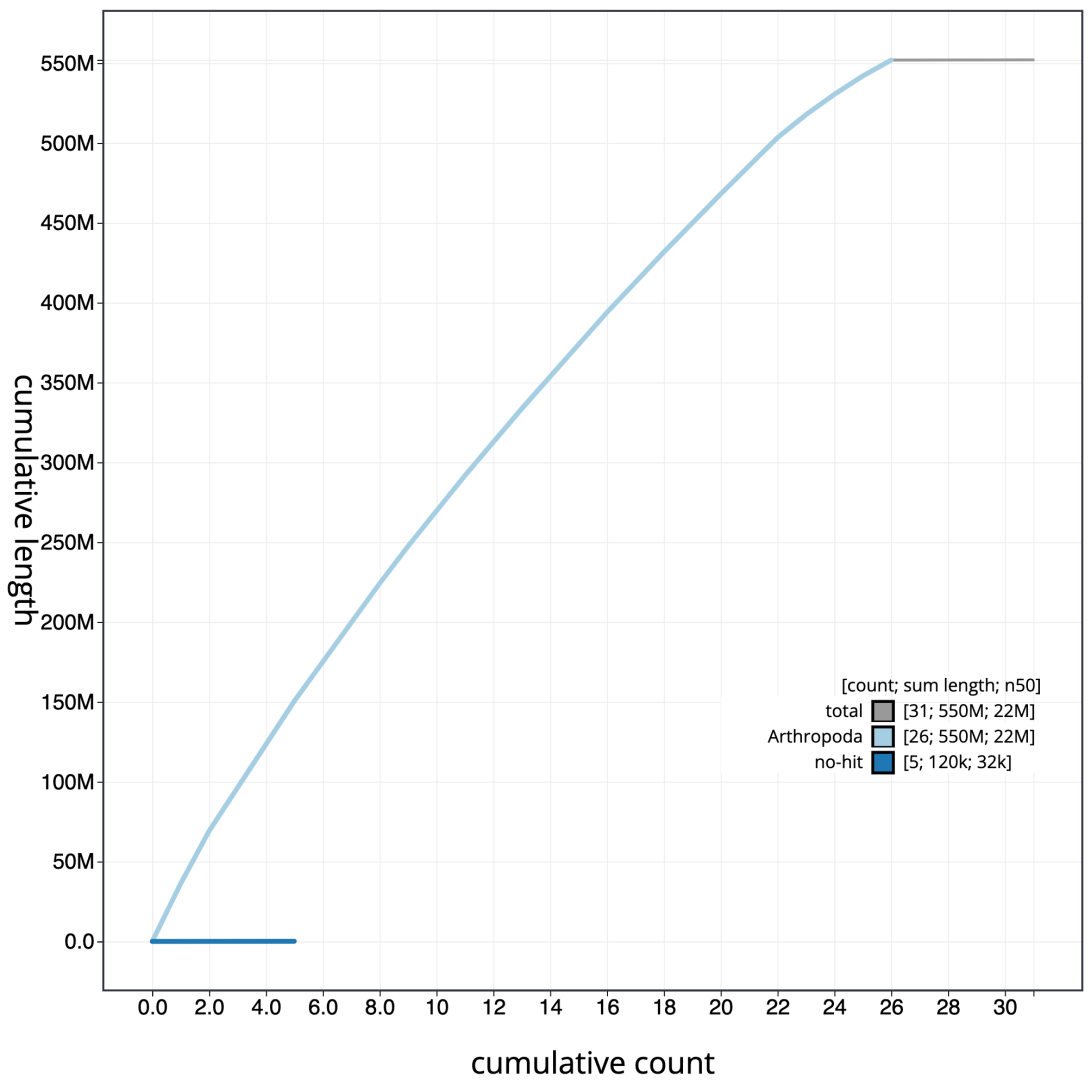
Genome assembly of *Incurvaria masculella*, ilIncMasc1.2: cumulative sequence. BlobToolKit cumulative sequence plot. The grey line shows cumulative length for all scaffolds. Coloured lines show cumulative lengths of scaffolds assigned to each phylum using the buscogenes taxrule. An interactive version of this figure is available at https://blobtoolkit.genomehubs.org/view/ilIncMasc1.2/dataset/CAMPPI02/cumulative.

**Figure 5.**
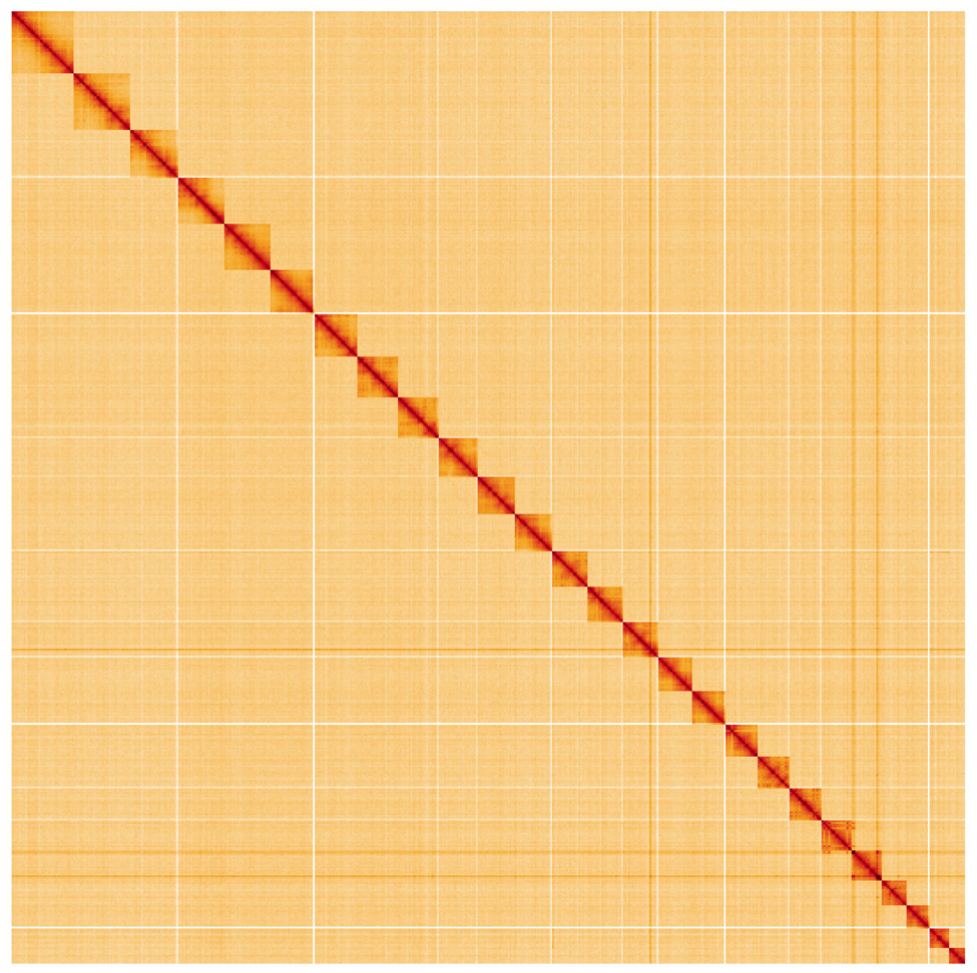
Genome assembly of *Incurvaria masculella*, ilIncMasc1.2: Hi-C contact map. Hi-C contact map of the ilIncMasc1.2 assembly, visualised using HiGlass. Chromosomes are shown in order of size from left to right and top to bottom. An interactive version of this figure may be viewed at https://genome-note-higlass.tol.sanger.ac.uk/l/?d=H2Dc25S1RqW8TtUHTYp_Xg.

**Table 2.** Chromosomal pseudomolecules in the genome assembly of *Incurvaria masculella*, ilIncMasc1.

INSDC accession	Chromosome	Size (Mb)	GC%
OX335641.1	1	36.25	39.1
OX335642.1	2	32.89	39
OX335643.1	3	27.36	39.2
OX335644.1	4	27.35	40.2
OX335645.2	5	24.85	39.8
OX335646.2	6	24.67	39.7
OX335647.2	7	24.16	39.9
OX335648.2	8	23.28	40
OX335649.1	9	22.33	39.1
OX335650.1	10	21.89	39.5
OX335651.1	11	21.18	39.3
OX335652.1	12	20.75	39.1
OX335653.1	13	20.25	39.3
OX335654.1	14	20.16	39.6
OX335655.1	15	19.94	39.9
OX335656.1	16	18.94	39.6
OX335657.1	17	18.81	39.6
OX335658.1	18	18.18	39.3
OX335659.1	19	18.17	40
OX335660.1	20	17.85	39.9
OX335661.1	21	17.5	40.5
OX335662.1	22	14.34	39.4
OX335663.1	23	12.75	40.3
OX335664.1	24	11.48	40.7
OX335665.1	25	9.95	40.4
OX335666.2	Z	26.65	39
OX335667.1	MT	0.02	19.6

## Methods

### Sample acquisition and nucleic acid extraction

A male *I. masculella* ( Figure 1) was collected from Wytham Woods, Oxfordshire (biological vice-country: Berkshire), UK (latitude 51.772, longitude –1.338) by William Langdon (University of Oxford) on 12 May 2021; this specimen (ToLID ilIncMasc1, specimen Ox001334) was used for acquisition of the genome sequence. A male *I. masculella* was found on a windowpane during daytime in Wallingford, Oxfordshire, UK (latitude 51.604, longitude –1.139) by Peter Holland (University of Oxford) on 9 May 2021; this specimen (ToLID ilIncMasc2, specimen Ox001335) was used for Hi-C scaffolding. Each specimen was identified by the collector and frozen at –80°C.

DNA was extracted at the Tree of Life laboratory, Wellcome Sanger Institute (WSI). The ilIncMasc1 sample was weighed and dissected on dry ice with tissue set aside for Hi-C sequencing. Whole organism tissue was disrupted using a Nippi Powermasher fitted with a BioMasher pestle. High molecular weight (HMW) DNA was extracted using the Qiagen MagAttract HMW DNA extraction kit. HMW DNA was sheared into an average fragment size of 12–20 kb in a Megaruptor 3 system with speed setting 30. Sheared DNA was purified by solid-phase reversible immobilisation using AMPure PB beads with a 1.8X ratio of beads to sample to remove the shorter fragments and concentrate the DNA sample. The concentration of the sheared and purified DNA was assessed using a Nanodrop spectrophotometer and Qubit Fluorometer and Qubit dsDNA High Sensitivity Assay kit. Fragment size distribution was evaluated by running the sample on the FemtoPulse system.

### Sequencing

Pacific Biosciences HiFi circular consensus DNA sequencing libraries were constructed according to the manufacturers’ instructions. DNA sequencing was performed by the Scientific Operations core at the WSI on Pacific Biosciences SEQUEL II (HiFi) instrument. Hi-C data were also generated from whole organism tissue of ilIncMasc2 using the Arima v2 kit and sequenced on the Illumina NovaSeq 6000 instrument.

### Genome assembly

Assembly was carried out with Hifiasm ( Cheng *et al.*, 2021) and haplotypic duplication was identified and removed with purge_dups ( Guan *et al.*, 2020). The assembly was scaffolded with Hi-C data ( Rao *et al.*, 2014) using YaHS ( Zhou *et al.*, 2023). The assembly was checked for contamination and corrected using the gEVAL system ( Chow *et al.*, 2016) as described previously ( Howe *et al.*, 2021). Manual curation was performed using gEVAL, HiGlass ( Kerpedjiev *et al.*, 2018) and Pretext ( Harry, 2022). The mitochondrial genome was assembled using MitoHiFi ( Uliano-Silva *et al.*, 2022), which performed annotation using MitoFinder ( Allio *et al.*, 2020). The genome was analysed and BUSCO scores generated within the BlobToolKit environment ( Challis *et al.*, 2020). Table 3 contains a list of all software tool versions used, where appropriate.

**Table 3.** Software tools and versions used.

Software tool	Version	Source
BlobToolKit	4.0.7	Challis *et al.*, 2020
gEVAL	N/A	Chow *et al.*, 2016
Hifiasm	0.16.1-r375	Cheng *et al.*, 2021
HiGlass	1.11.6	Kerpedjiev *et al.*, 2018
MitoHiFi	2	Uliano-Silva *et al.*, 2022
PretextView	0.2	Harry, 2022
purge_dups	1.2.3	Guan *et al.*, 2020
YaHS	yahs-1.1.91eebc2	Zhou *et al.*, 2023

### Ethics and compliance issues

The materials that have contributed to this genome note have been supplied by a Darwin Tree of Life Partner. The submission of materials by a Darwin Tree of Life Partner is subject to the Darwin Tree of Life Project Sampling Code of Practice. By agreeing with and signing up to the Sampling Code of Practice, the Darwin Tree of Life Partner agrees they will meet the legal and ethical requirements and standards set out within this document in respect of all samples acquired for, and supplied to, the Darwin Tree of Life Project. All efforts are undertaken to minimise the suffering of animals used for sequencing. Each transfer of samples is further undertaken according to a Research Collaboration Agreement or Material Transfer Agreement entered into by the Darwin Tree of Life Partner, Genome Research Limited (operating as the Wellcome Sanger Institute), and in some circumstances other Darwin Tree of Life collaborators.

## Data Availability

European Nucleotide Archive: *Incurvaria masculella*. Accession number PRJEB55135; https://identifiers.org/ena.embl/PRJEB55135 ( Wellcome Sanger Institute, 2022) The genome sequence is released openly for reuse. The *Incurvaria masculella* genome sequencing initiative is part of the Darwin Tree of Life (DToL) project. All raw sequence data and the assembly have been deposited in INSDC databases. The genome will be annotated using available RNA-Seq data and presented through the Ensembl pipeline at the European Bioinformatics Institute. Raw data and assembly accession identifiers are reported in Table 1.

## Author information

Members of the University of Oxford and Wytham Woods Genome Acquisition Lab are listed here: https://doi.org/10.5281/zenodo.4789928.

Members of the Darwin Tree of Life Barcoding collective are listed here: https://doi.org/10.5281/zenodo.4893703.

Members of the Wellcome Sanger Institute Tree of Life programme are listed here: https://doi.org/10.5281/zenodo.4783585.

Members of Wellcome Sanger Institute Scientific Operations: DNA Pipelines collective are listed here: https://doi.org/10.5281/zenodo.4790455.

Members of the Tree of Life Core Informatics collective are listed here: https://doi.org/10.5281/zenodo.5013541.

Members of the Darwin Tree of Life Consortium are listed here: https://doi.org/10.5281/zenodo.4783558.
