# Supplementary material for: The genome sequence of the Feathered Bright, Incurvaria masculella (Denis & Schiffermüller, 1775)
Source: Wellcome Open Res. 2023 Mar 30;8:144. [Version 1] doi: 10.12688/wellcomeopenres.19205.1 (PMC10682600; doi:10.12688/wellcomeopenres.19205.1)
Supplement: Peer review history

# Reviewer report: Reviewer response for version 1

**DOI:** 10.21956/wellcomeopenres.21287.r69256

**Published:** 2023-11-27

**Authors:** James Mallet

**Affiliations:** 1 Department of Organismic and Evolutionary Biology, Harvard University, Cambridge, USA

This paper concerns an assembled genome of a moth (Lepidoptera) in the family Incurvariidae. Most studies of Lepidoptera, including genetic studies, are done in the large crown group, Ditrysia. However, the other groups of Lepidoptera, such as the Incurvariidae, will enable a better understanding of evolution of the Lepidoptera as a whole, so the current genome assembly is going to be a welcome addition to Lepidoptera genomics.

 I am very approving of the release of the genome for open re=use. 

 I would have thought the authors could comment on the synteny of the 26 chromosomes identified at least in a somewhat superficial way. A number of studies have shown that Lepidoptera chromosomes are strongly conserved with a basal number of 31 across the whole order. Thus, unless *Incurvaria *is exceptional (like some Pieridae), one would expect around 5 simple fusions from the ancestral karyotype to explain the *Incurvaria *n = 26. Is this the case?

 The only indication of synteny interest I could find in the draft paper was "The Z chromosome was identified by similarity." -- but similarity to what, exactly?

 "BUSCO v5.3.2 completeness of 91.9% (single 91.1%, duplicated 0.8%) using the lepidoptera_odb10 reference set." This seems good, but unexpectedly low for a well-sequenced genome. However, I assume that the BUSCO orthologs are largely based on Ditrysians, and maybe the reduced BUSCO score achieved here is due to the standard not really applying so well to this more "basal" lineage.

 Overall, I applaud this effort to gain excellent genome assemblies of obscure taxa and hope my comments are not discouraging. Maybe a few more quick pointers to the interest in the particular species and data may help generate even more interest.

Are sufficient details of methods and materials provided to allow replication by others?

Yes

Is the rationale for creating the dataset(s) clearly described?

Yes

Are the datasets clearly presented in a useable and accessible format?

Yes

Are the protocols appropriate and is the work technically sound?

Yes

Reviewer Expertise:

Lepidoptera evolution and genomics

I confirm that I have read this submission and believe that I have an appropriate level of expertise to confirm that it is of an acceptable scientific standard.

# Reviewer report: Reviewer response for version 1

**Published:** 2023-11-06

**Authors:** Craig Michell

**Affiliations:** 1 BESE, King Abdullah University of Science and Technology, Thuwal, Makkah Province, Saudi Arabia

**Summary:**

 The authors here present a high-quality genome of two males (One for the primary genome and another for Hi-C) from the species *Incurvaria masculella* (Denis & Schiffermüller, 1775). The reported genome is 552 Mbp in length has a high contiguity and level of completeness. The genome will provide an excellent resource for further studies into the evolution of Lepidoptera.

**Methods:**

 The authors used appropriate methods to assemble the genome of this species. The technologies used for the genome assembly include high genome coverage PacBio HiFi sequencing, and Hi-C sequencing.

**Datasets:**

 The datasets presented are clear and freely accessible online. The genome seems to be well assembled and the metrics are presented clearly.

**Comments:**

 I have only very minor comments regarding the manuscript as everything is clear and the data presented is logical and freely accessible.

1. “This sex-specific difference suggests that the unusual comb-like shape of the male antenna is likely an adaptation for increased olfactory reception in mate-finding” This is a relatively strong statement, do you have a reference to back this up?
2. "The assembly was scaffolded with Hi-C data ( Rao *et al.*, 2014)" this is ambiguous as it would suggest that the Hi-C data came from Rao *et al* 2014. This is not the case. I am not sure what it could be referring to. The library was produced using the Armina kit, and the scaffolding was done using YaHS. If it is a citation for Hi-C in general then the sentence needs to be reworded.
3. In terms of the tools used, all are cited, however BUSCO which is used through blobtools should also be cited.

Are sufficient details of methods and materials provided to allow replication by others?

Yes

Is the rationale for creating the dataset(s) clearly described?

Yes

Are the datasets clearly presented in a useable and accessible format?

Yes

Are the protocols appropriate and is the work technically sound?

Yes

Reviewer Expertise:

Genome assembly of non-model organisms

I confirm that I have read this submission and believe that I have an appropriate level of expertise to confirm that it is of an acceptable scientific standard.
